# Thermal Release Transfer Printing for Stretchable Conformal Bioelectronics

**DOI:** 10.1002/advs.201700251

**Published:** 2017-07-31

**Authors:** Zhuocheng Yan, Taisong Pan, Miaomiao Xue, Changyong Chen, Yan Cui, Guang Yao, Long Huang, Feiyi Liao, Wei Jing, Hulin Zhang, Min Gao, Daqing Guo, Yang Xia, Yuan Lin

**Affiliations:** ^1^ State Key Laboratory of Electronic Thin Films and Integrated Devices University of Electronic Science and Technology of China (UESTC) Chengdu Sichuan 610054 P. R. China; ^2^ Key Laboratory for NeuroInformation of Ministry of Education School of Life Science and Technology University of Electronic Science and Technology of China (UESTC) Chengdu Sichuan 610054 P. R. China; ^3^ Center for Information in BioMedicine University of Electronic Science and Technology of China (UESTC) Chengdu Sichuan 610054 P. R. China

**Keywords:** brain–computer interface (BCI), neural electrode arrays, stretchable electronics, transfer printing

## Abstract

Soft neural electrode arrays that are mechanically matched between neural tissues and electrodes offer valuable opportunities for the development of disease diagnose and brain computer interface systems. Here, a thermal release transfer printing method for fabrication of stretchable bioelectronics, such as soft neural electrode arrays, is presented. Due to the large, switchable and irreversible change in adhesion strength of thermal release tape, a low‐cost, easy‐to‐operate, and temperature‐controlled transfer printing process can be achieved. The mechanism of this method is analyzed by experiments and fracture‐mechanics models. Using the thermal release transfer printing method, a stretchable neural electrode array is fabricated by a sacrificial‐layer‐free process. The ability of the as‐fabricated electrode array to conform different curvilinear surfaces is confirmed by experimental and theoretical studies. High‐quality electrocorticography signals of anesthetized rat are collected with the as‐fabricated electrode array, which proves good conformal interface between the electrodes and dura mater. The application of the as‐fabricated electrode array on detecting the steady‐state visual evoked potentials research is also demonstrated by *in vivo* experiments and the results are compared with those detected by stainless‐steel screw electrodes.

## 1 Introduction

Neurophysiologic monitoring is commonly used for diagnosing and treating neurological disorders such as epilepsy, Parkinson's disease, Alzheimer's disease, depression, and disorders of the peripheral nervous system.^1^ In neurophysiologic monitoring techniques, electroencephalography (EEG) or electrocorticography (ECoG), with the ability to monitor brain function changes with high spatiotemporal resolution, is the gold standard for positioning epileptic foci and brain function areas.^2^ As the core of neurophysiologic monitoring, realizing high‐quality signal collection is an important topic in the research of neural electrode arrays. Related researches have indicated that stretchable/flexible neural electrode arrays, with their excellent mechanical and electrical performance, are superior candidates for the next generation of implantable devices.^3^, ^4^, ^5^, ^6^, ^7^, ^8^, ^9^, ^10^, ^11^ The successful development of stretchable/flexible neural electrode arrays hinges on good conformal contact between the array and the tissue, which enables excellent mechanical and electrical properties,^3^ as well as low‐cost, stable, and high‐throughput manufactural techniques.

With the advantages for highly accurate and highly compatibility assembly of micro/nanostructured materials on elastomers, the transfer printing technique is considered to be a feasible solution for the fabrication of stretchable/flexible neural electrode arrays.^12^ The transfer printing technique involves the use of a soft elastomeric stamp to transfer solid micro/nanostructured materials from a donor substrate onto a receiver substrate for device integration.^13^ In this technique, dynamic control of the interfacial adhesion between the stamp and the object to be transferred plays a crucial role in completing successful transfer printing. As shown in **Table** **1**, several strategies for adhesion control of transfer printing technique have been proposed and applied in the stretchable bioelectronics fabrication (e.g., complex 3D mesostructures,^14^, ^15^, ^16^, ^17^, ^18^, ^19^, ^20^ wireless biomedical devices,^17^, ^21^, ^22^, ^23^, ^24^, ^25^, ^26^, ^27^ and epidermal sensor systems^23^, ^28^, ^29^, ^30^, ^31^, ^32^, ^33^, ^34^, ^35^).

**Table 1:** List of transfer printing methods

Strategies	Mechanism	References
Kinetic control	Adjusting the viscoelasticity of stamps at different peeling rates	13, 36, 37, 38, 39
Surface‐relief‐assisted control	Changing contact area between stamp surface and microdevices	40, 41, 42, 43
Load‐enhanced control	Utilizing the mechanical loading of stamps to modulate adhesion strengths	44, 45, 46
Laser‐driven control	Inducing large thermal mismatch between the stamp and microdevices	47, 48
Shape memory driven control	Adopting shape memory effect of stamps to manipulate reversible dry adhesion via temperature change	49, 50, 51

However, some drawbacks of current adhesion control strategies also emerge. The tunability of the kinetic control strategy based solely on the peeling velocity‐dependent adhesion of elastomeric stamps is quite limited. In order to increase the strong to weak adhesion ratio, additional lithography processes associated with modifying stamps by inducing relief microstructures on the stamp surface may be employed. These may impact the cost and convenience in the manufacture of stretchable bioelectronics. Moreover, many implementations of stretchable/flexible bioelectronics employ polyimide (PI), a polymer with excellent mechanical properties and thermostability,^1^ as a flexible passivation layer to reinforce the metal layers.^52^ The insufficient strong to weak adhesion ratio of kinetic control strategy requires an additional thin layer of polymethyl methacrylate (PMMA) as a sacrificial layer on the donor substrate before the fabrication of the bioelectronics to decrease the interfacial adhesion between the bioelectronics membrane and the substrate.^5^, ^35^, ^53^, ^54^ Extra processing steps related to the fabrication and removal of the sacrificial layer increase the complicacy of the process. Besides, the conventional thick stamp (e.g., shape memory polymer, polydimethylsiloxane (PDMS), or microstructured elastomer) used for picking up is hard to be compatible with the roll‐to‐roll process.

Here, we propose a thermal release transfer printing approach for stretchable bioelectronics manufacture, which is expected to provide a solution to the challenges mentioned above. In this approach, a thermal release tape (TRT) is used as a stamp to replace the conventional thick elastomer stamp. TRT is a kind of thin, flexible and roll‐to‐roll compatible tape, which shows large, switchable and irreversible change in adhesion strength when it is heated to around 100 °C. Although several successful applications of TRT in transferring monolayer 2D materials (e.g., graphene^55^, ^56^, ^57^ or MoS_2_ 58), ink,^59^ and silicon^60^ have been demonstrated by controlling adhesion strength of TRT via varying the temperature, the principle of thermal release transfer printing with TRT has not been discussed in details. In this study, the mechanism of this approach is analyzed by studying the peeling velocity and temperature dependence of energy release rate with experiments and fracture‐mechanics models. The models demonstrate that thermal released transfer printing method has a larger strong to weak adhesion ratio than the kinetically controlled transfer printing method. By utilizing the large strong to weak adhesion ratio of TRT, the metal/PI based microdevices can be picked up directly from PI/glass interface using a sacrificial‐layer‐free process, and printed onto a PDMS substrate by thermal treatment. Due to the small Van der Waals force between the conventional PDMS stamp and microdevices, the retrieval of the metal/PI‐based microdevices cannot be accomplished by the common kinetically controlled transfer printing method if no sacrificial layer is fabricated.^5^, ^54^ Using the thermal release transfer printing approach, a stretchable neural electrode array on ultrathin elastomeric substrate has been designed and fabricated. By theoretically and experimentally evaluating the contact performance of the electrodes with various elastomeric substrate thicknesses, good conformal contact is realized when using a substrate thinner than the calculated critical thickness. The *in vivo* ECoG measurement experiments on anesthetized rats have demonstrated the feasibility of the design and the fabrication method. The levels of fidelity in ECoG signals that detected by the as‐fabricated neural electrode array with an optimized substrate thickness are comparable with or even better than those detected by standard stainless‐steel screw electrodes. The application of the as‐fabricated electrode array on detection of steady‐state visual evoked potentials (SSVEP) response has also been demonstrated by *in vivo* experiments and the results have been compared with stainless‐steel screw electrodes.

## 2 Results and Discussion

**Figure 1:**
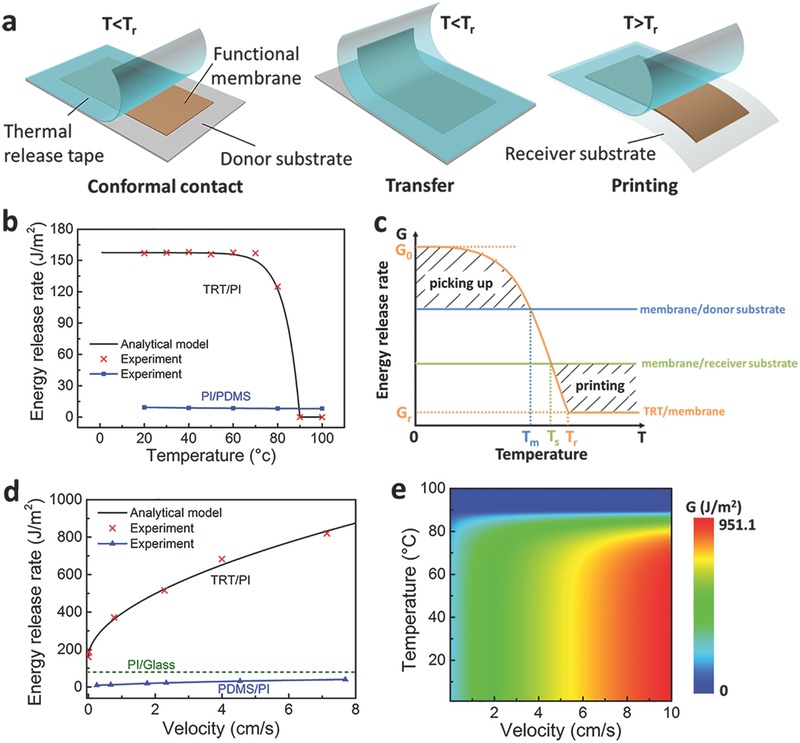
The mechanism of thermal release transfer printing process. a) Schematic illustration of thermal release transfer printing method. b) Experimental data and theoretical analysis' results of the relationship between the temperature and energy release rate of TRT/PI and PI/PDMS interface. c) Schematic diagram showing the change of TRTs' energy release rate by controlling temperature. d) Experimental data and theoretical analysis' results of the relationship between the velocity and the energy release rate of TRT/PI and PDMS/PI interface. The dotted line represents the energy release rate of PI/Glass interface. e) The contour map showing the relationship between velocity, temperature and energy release rate of TRT/PI interface.

**Figure** **1**a illustrates the general process of thermal release transfer. The process starts from the conformal contact between the functional membrane and TRT. After TRT is bonded with the top surface of membrane, the functional membrane to be transferred is peeled away from the donor substrate by the adhesion force of bonded TRT. Then the functional membrane on the TRT is transferred to the receiver substrate by contacting the membrane with the substrate. A following heating step to a temperature higher than the transition temperature (*T*_r_) weakens the adhesion between the membrane and TRT, leading to the separation of TRT from the membrane. Then, the as‐transferred membrane is “printed” on the receiver substrate and the thermal release transfer process is completed. It can be seen from the processes that the core of the process is to finish the transfer and printing by adjusting the adhesion property of stamp. Successful thermal transfer release highly depends on the changing of the adhesion property between the membrane and TRT from a strong one to a weak one. As the adhesion strength of TRT can be adjusted by a transition temperature *T*_r_ (i.e., the melting point), the adhesion behavior of TRT is a temperature‐dependent two‐stage one. When the temperature increases to be higher than *T*_r_, the weakened adhesion strength of TRT makes it be easily removed from the membrane bonded at a low temperature (<*T*_r_). The thermal transfer release process can be modeled by two competing fracture paths that have different energy release rates (*G*): the TRT/membrane interface and the membrane/substrate interface. The energy release rate *G* for these fracture paths can be described as steady‐state crack propagation, which can be determined by^13^

(1) $$G=\frac{F}{w}$$

where *F* is the peeling force, *w* is the width of the interface. According to the Griffith criterion, *G* describes the energy of interfacial bond breaking and adhesive dissipation around the crack tip, and the crack will propagate steadily when the value of *G* reaches the critical *G* value. After denoting the critical *G* value for the membrane/donor substrate, membrane/receiver substrate and the TRT/membrane interface by *G*_membrane/donor substrate_ *_,_ G*_membrane/receiver substrate_, and *G*_TRT/membrane_, respectively, the picking up and printing process can be described by the relation between critical energy release rates *G* of these three interfaces. To successfully pick up the functional membrane from the donor substrate, *G*_TRT/membrane_ is required to be larger than *G*_membrane/donor substrate_. In other words, the crack will start to propagate in the membrane/donor substrate interface first. Conversely, if *G*_TRT/membrane_ is smaller than *G*_membrane/receiver substrate_, the crack will initiate and propagate at the TRT/membrane interface, i.e., TRT will be peeled off from the bonded functional membrane and the functional membrane will be “printed” on the receiver substrate.

As *G*_membrane/donor substrate_ and *G*_membrane/receiver substrate_ depend on the material properties of the membrane/donor substrate and membrane/receiver substrate interfaces and are stable for most material combinations, the key of thermal transfer release process is to control the critical energy release rate of the TRT/membrane interface (*G*_TRT/membrane_). Since the adhesion of TRT depends on temperature, *G*_TRT/membrane_ is temperature related. The temperature dependency of *G*_TRT/membrane_ is experimentally measured at the temperature ranging from 20 to 100 °C, in a system with PI as the membrane, as indicated by Figure 1b. It can be seen that *G*_TRT/membrane_ significantly decreases when the temperature is higher than about 80 °C. Based on the experimental results, an analytical model is proposed for the relation between the temperature and *G*_TRT/membrane_

(2) $$\begin{array}{l} G_{\mathrm{TRT/membrane}}\left(T\right)\\ =\left\{ \begin{array}{l} -e^{r\left(T-T_{r}\right)\;+\;\ln\left(G_{0}-G_{r}\right)}+G_{0}\;\;\;\;T\leq T_{r}\\ G_{r}T>T_{r} \end{array}\right. \end{array}$$

where *T* is the current temperature, *e* is the Euler's constant, *G*_0_ is the initial critical energy release rate, *G*_r_ is the critical energy release rate when the adhesives on TRT is deactivated. *T*_r_ is the transition temperature above which the adhesive force of TRT rapidly reduces, γ is a parameter depending only on the compositions of materials. When *G*_0_ = 157.5 J m^−2^, *G*_r_ = 0 J m^−2^, γ = 0.15, *T*_r_ = 90 °C, the relation between the temperature and *G*_TRT/membrane_ is plotted in Figure 1b and shows good agreement with the experimental data. Moreover, the relation between the critical energy release rate of the PI/PDMS interface (*G*_PI/PDMS_) and the temperature is also shown in Figure 1b. It is seen that the *G*_PI/PDMS_ does not change obviously in the temperature range from 20 to 100 °C. When the temperature is higher than 90 °C, due to that *G*_TRT/PI_ < *G*_PI/GPDMS_, the PI membrane can be released from TRT to the PDMS substrate. Therefore, PDMS can be used as a receiver substrate in the thermal release transfer printing method.

As shown in Figure 1c, the entire thermal transfer release process can be separated into “picking up” and “printing” parts by the temperature point *T*_m_ and *T*_s_. When the temperature is lower than *T*_m_, *G*_TRT/membrane_ is larger than critical energy release rate of the membrane/donor substrate interface, *G*_membrane/donor substrate_, the crack prefers to propagate at the membrane/donor substrate interface, which makes the membrane be easily picked up from donor substrate. After transferring the membrane onto the surface of the target substrate with TRT, heating the target substrate will lead to decrease of *G*_TRT/membrane_. When the temperature is higher than *T*_s_, *G*_TRT/membrane_ will be smaller than the critical energy release rate of the membrane/receiver substrate interface, *G*_membrane/receiver substrate_. In this circumstance, peeling off TRT leads to crack propagation at the TRT/membrane interface and the membrane is “printed” on the target substrate.

The discussion on the role of energy release rate in the thermal transfer release process provides an insight into the mechanical nature of thermal transfer release process. From the perspective of energy release rate, we can also see the advantage of thermal transfer release process. Being similar with the thermal transfer release process, conventional kinetically controlled transfer printing is dominated by adjusting energy release rate of stamp/membrane interface with changing peeling speed of stamp. The critical energy release rate of the interface between the membrane and the popular PDMS stamp used in kinetically controlled transfer printing, which can be obtained from experimental results in Figure 1d and Figure S1 (Supporting Information), is much smaller than the critical energy release rate of TRT/membrane interface. The large critical energy release rate makes it possible to use TRT to directly pick up functional membranes from donor substrates with a large *G*_membrane/donor substrate_ (Figure S2, Supporting Information). From the example in Figure 1d, in a system with PI as the membrane, the critical energy release rate of the PI/Glass interface (*G*_PI/Glass_) is about 79.7 J m^−2^, obtained from peel test (Figure S3, Supporting Information). As the *G*_PI/Glass_ is always smaller than *G*_TRT/PI_ and larger than *G*_PDMS/PI_ when the peeling velocity ranges from 0 to 7.8 cm s^−1^, the TRT is able to pick up PI directly from glass substrate but PDMS is unable to. In other words, it is necessary to coat PMMA as a sacrificial layer to decrease the interfacial adhesion between PI and donor substrate for the kinetically controlled transfer printing using PDMS as a stamp, whereas transfer printing with the thermal transfer release process using TRT as a stamp can avoid the preparation of a sacrifice layer between the donor substrate and the functional membrane.

The influence of peeling velocity on the critical energy release rate *G*_TRT/membrane_ has also been carefully studied. The relation between *G*_TRT/PI_ and peeling velocity measured at 20 °C is plotted in Figure 1d. It is observed that a larger critical energy release rate *G*_TRT/membrane_ can be obtained by increasing the peeling velocity. Faster peeling of TRT makes the separation between PI and the donor substrate easier. The investigation shows that *G*_TRT/membrane_ can be considered to be determined by two parameters, temperature, and peeling velocity. Then, Equation (3) can be further developed as

(3) $$\begin{array}{l} G_{\mathrm{TRT/membrane}}\left(v,T\right)\\ =\left\{ \begin{array}{l} \left(-e^{\gamma \left(T-T_{r}\right)\;+\;\ln\left(G_{0}-G_{r}\right)}+G_{0}\right)\left(1+{\left(\frac{v}{v_{0}}\right)}^{\;n}\right)\;\;\;\;\;\;T\leq T_{r}\\ G_{r}\left(1+{\left(\frac{v}{v_{0}}\right)}^{\;n}\right)T>T_{r} \end{array}\right. \end{array}$$

where *v* is current peeling velocity, *v*_0_ is the reference velocity, *n* is an undetermined parameter, which can be calculated by fitting experimental data. As indicated by Figure 1d, when *v*_0_ = 0.5 cm s^−1^, *n* = 0.54, the result of the analytical model and experiments are consistent. According to Equation (3), a contour plot (Figure 1e) can be used to illustrate the relation among peeling velocity of TRT, temperature, and critical energy release rate. It can be seen from Figure 1e that the critical energy release rate *G*_TRT/membrane_ can be controlled in a wide range by adjusting the peeling velocity of TRT and the temperature in the thermal transfer release process. The wide‐range adjustable *G*_TRT/membrane_ provides the ability to transfer functional membranes from donor substrates with different critical energy release rates *G*_membrane/donor substrate_. Furthermore, the TRT used in this method can pick up microdevices much easier than kinetically controlled transfer printing due to its larger strong to weak adhesion ratio.

Moreover, the applicability of Equations (2), (3) is also validated in TRT/polyethylene terephthalate (PET) system. The plots in Figure S4a,b indicate that when change the membrane from PI to PET, the result of experiments is also consistent with the corresponding analytical models (Equation (3); Equation S7, Supporting Information). These analyses clearly show that the proposed theoretical model in this study is applicable to not only TRT/PI interface, but the interface involving TRT and other materials.

**Figure 2:**
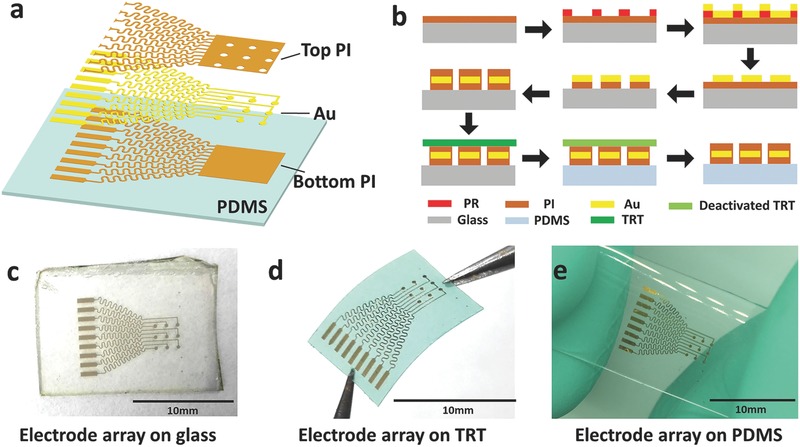
Fabrication of a neural electrode array using the thermal release transfer printing method. a) Exploded view schematic diagram of the electrode array layout. b) Schematic illustration of the fabrication process for transferable neural electrode array on PDMS using the thermal release transfer printing method. c) Transferable stretchable neural electrode array made on glass. d) Picking up the stretchable neural electrode array by TRT. e) The neural electrode array printed on the PDMS substrate after the thermal treatment and peeling off the TRT.

By using the thermal transfer release process, a stretchable neural electrode array is designed and fabricated. **Figure** **2**a shows the schematic illustration of a stretchable neural electrode array consisting of nine channels with a three‐layered serpentine‐like interconnect structure. An 80 nm thick Au layer encapsulated with PI (1 µm thick on the top and 0.7 µm on the bottom) serves as the conductive layer to collect the ECoG signal. The layout of the Au conductive layer includes three parts: the output part, the interconnect part, and the input part. The output part is designed as nine parallel square pads to connect to the anisotropic conductive film (ACF) cable of ECoG recorder. The interconnectors are serpentine lines with 100 µm in width. Nine small circles, serving as the input part, are connected to the output part via the serpentine interconnectors. The PI layer on the top of the output part (square pad) and the input part (circles) are removed to provide electrical connectivity.

A schematic of the fabrication procedures of such a device using the thermal release transfer process is illustrated in Figure 2b. The fabrication starts from spin‐coating a layer of polyimide onto a glass slide at 4000 rpm to make the bottom layer of electrodes. After amination at 180 °C for 2 h, an Au layer with the designed pattern is deposited on the PI layer using the standard lift‐off technique. Then the bottom PI is etched by 0.5% sodium hydroxide solution (NaOH) to obtain the micron/nanosized pattern. After that, another layer of polyimide is spin‐coated at 3000 rpm and aminated at 120 °C for 20 min in order to serve as the top layer. The top PI layer can be patterned using the photolithography process. After photolithography and developing of the top PI layer, the electrode array is obtained on the glass slide. Before transfer printing, the elastomeric substrate (PDMS) is preprocessed in ultraviolet light for 2 min in order to clean and activate its surface. Then the electrode array can be picked up directly by TRT and printed to PDMS substrate by a heating step. As can be seen in Figure 2c–e, the as‐prepared PI/Au/PI membrane is in good condition during the entire thermal transfer release process (more detailed images can be seen in Figures S5 and S6, Supporting Information and Supporting Information video 1). The integrity of the transferred micron/nanosized devices on elastomeric substrates attests to the feasibility of the thermal release transfer printing method.

**Figure 3:**
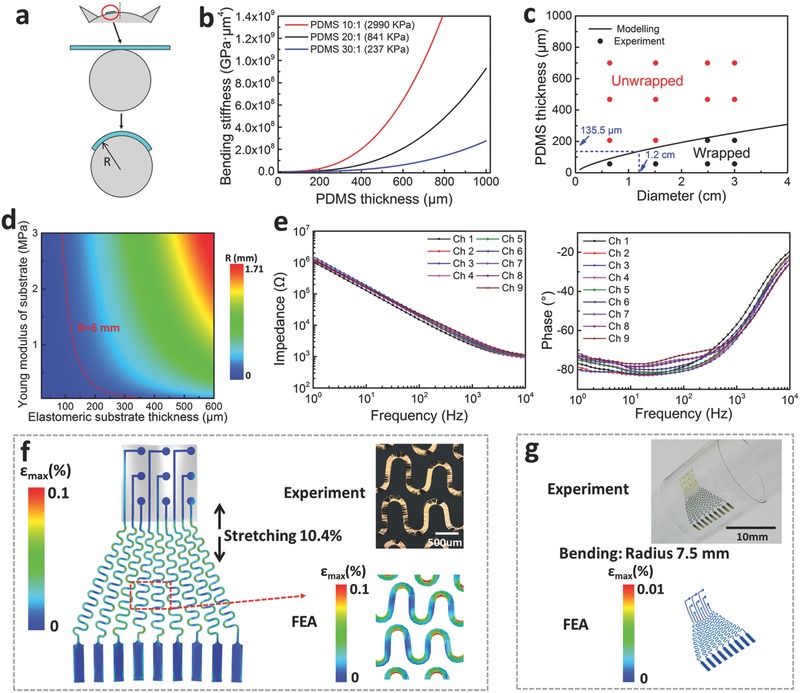
Mechanical modeling and electrical/mechanical characteristics of the electrode. a) The mechanics model for a thin film wrapping around a rat brain and a cylinder of radius *R*. b) Total bending stiffness of stretchable neural electrode arrays on PDMS substrates of the different mixing ratio as a function of the PDMS thickness. c) Comparing results from the mechanics modeling and wrapping experiments. d) The contour map showing the relationship between the conformal critical radius (*R*), thickness and Young's modulus of the elastomeric substrate. e) Electrochemical impedance spectra, magnitude (left) and phase (right), measured at nine different recording sites in the stretchable neural electrode array configured for ECoG. f) The FEA strain distribution results of the electrodes arrays when under 10.4% uniaxial stretching along the vertical direction. g) The FEA strain distribution results of the electrodes arrays when bending to a radius of curvature of 7.5 mm.

Neural interfaces have to be mechanically compliant to promote conformal contact in order to accurately detect ECoG signals from the brain. As shown in **Figure** **3**a, the conformal contact issue of neural electrode array can be simplified as how to keep a membrane wrapping around a cylinder with certain radius. The formula of the conformal critical diameter is shown as the following^5^

(4) $$\gamma \geq \gamma_{c}=\frac{EI}{R^{2}b}$$

where γ is the adhesion energy per unit area, *b* is the width of PI pad (*b* = 3690 µm, Figure S7, Supporting Information). γ is about 10 mJ m^−2^, according to the reported values for wet interfaces.^5^ According to Equation (4), the total bending stiffness, EI, is the key variable in conformal contact issue. The detail calculations of EI are illustrated in Supporting Equations S1–S3 and Figure S7 (Supporting Information). The EI of a stretchable neural electrode majorly depends on the Young's modulus and the thickness of the elastomeric substrate. As shown in Figure 3b, EI of the electrode will quickly increase with increase of substrate thickness (Equations S1 and S2, Supporting Information). According to previous study,^61^ the Young's modulus of PDMS with mixing ratios of 10:1, 20:1, 30:1, are 2900, 841, and 237 KPa, respectively. With the same thickness of substrate, the EI of the electrode with a higher Young's modulus is larger than that with a lower Young's modulus. The values of the critical adhesion energy, γ_c_ for the neural electrode array (with the substrate of 20:1 PDMS) wrapping around cylinders of different radiuses are calculated according to Equation (4), as shown in Figure 3c. When γ ≥ γ_c_, neural electrode array would wrap around the cylinder. The corresponding experimental results (details shown in Figure S8, Supporting Information) demonstrate that reducing the substrate thickness provides clear benefits. For example, it is possible to wrap a cylinder with *R* = 6 mm using only capillary adhesion forces when the thickness of PDMS is less than 135.5 µm. In other words, due to the minimum bending radius of curvature of a rat brain is 6 mm,^1^ it is possible to enhance conformal contact between the electrode and the brain surface when the thickness of the device is less than 135.5 µm with the substrate of 20:1 PDMS. Furthermore, according to Equation (4), a contour plot (Figure 3d) can illustrate the relationship between the Young's modulus, the thickness of the elastomeric substrate, and the wrapping radius. For a certain radius surface to be wrapped, the conformal contact can be improved by adjusting the Young's modulus and the thickness of the elastomeric substrate. Therefore, based on the above analysis, the PDMS with 50 µm in thickness and 841 KPa of Young's modulus is used as the substrate of neural electrode to collect ECoG signals in our experiments.

The electrical and mechanical properties of the as‐fabricated electrode array have been experimentally evaluated. As the impedance of the electrode array is closely related to the noise level and the signal‐to‐noise ratio (SNR) of the recorded signals,^62^ the frequency dependence of electrode impedance has been measured. The plots in Figure 3e illustrate the impedance of each channel in array. Magnitude and phase of the impedance for each channel is at the same level with the previous reported experiment result of reported Au electrode,^8^ indicating the ability for multichannel electrical activity recording of the stretchable neural electrode. The impedance spectra after 10.4% applied strain and 100 cyclic stretching (10.4% applied strain) are also measured, which are shown in Figures S9 and S10 (Supporting Information). The results illustrate that there is no obvious change in impedance magnitude and phase of the device after 100 cyclic stretching with 10.4% applied strain. The mechanical properties of the stretchable neural electrode have been analyzed with experiments and finite element analysis (FEA). As shown in Figure 3f, according the strain distribution in the Au layer derived from the FEA results, no elastic–plastic transition occurs when the substrate is stretched to 10.4%. The images in Figure S11 (Supporting Information) of as‐fabricated electrode array under cyclic stretching from 0% to 10.4% for 2000 times indicate that no apparent cracks have been observed on it, which is consistent with the FEA result of the strain distribution in the Au film layer. The zoomed‐in deformation of electrode obtained by FEA shown in Figure 3f is also consistent with the optical image of stretched electrode. FEA results shown in Figure 3g corresponding to experimental result demonstrate that the strain in the Au layer does not reach the yield strain (≈0.3%) upon bending on a rigid cylinder with a radius of ≈7.5 mm. These experiment and FEA results indicate that the mechanical properties of as‐fabricated electrode array facilitate the use of such a device on the tissue and brain.

**Figure 4:**
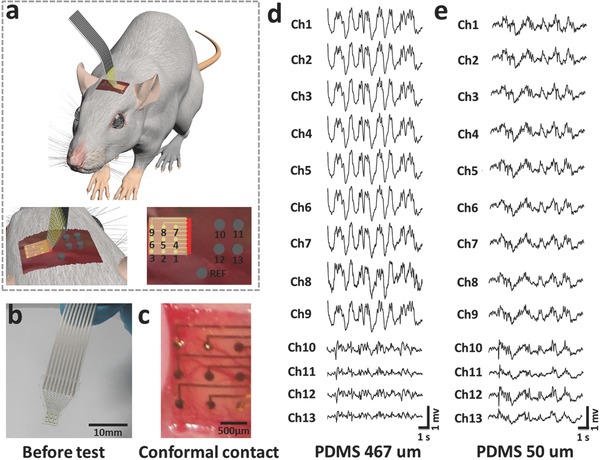
Photographs and data from murine validation experiments. a) The anatomy and locations of the stretchable neural electrode arrays relative to the visual cortex. Schematic illustration of nine channels of stretchable neural electrode array, four channels of stainless‐steel screw electrode, and one channel of reference electrode. b) Images of electrode array after connection with heat seal connector. c) Photograph of a nine‐channel stretchable neural electrode array placed on the visual cortical surface of the left hemisphere of a rat. d) Representative ECoG signals recorded by the stretchable neural electrode array with a 467 µm thick PDMS substrate (channels 1–9) and stainless‐steel screw electrodes (channels 10–13). e) Representative ECoG signals recorded by stretchable neural electrode array with a 50 µm thick PDMS substrate (channels 1–9) and stainless‐steel screw electrodes (channels 10–13).

**Figure** **4** displays ECoG measurement from anesthetized rats using UEA‐FZ amplifier (SYMPTO Company, Beijing, China). As shown in Figure 4a, two kinds of electrodes have been used for comparison: four channels with stainless‐steel screw electrodes (diameter, 500 µm) and nine channels with stretchable neural electrode array. All the electrodes are attached to dura mater. The rectangle window (4 × 4.5 mm) for the stretchable neural electrode array and the locations of the stainless‐steel screw electrodes are in symmetric visual cortex area. Figure 4b and Figure S12 (Supporting Information) indicate the good connection of heat seal connector (HSC) between the as‐fabricated stretchable neural electrode array and the printed circuit board (PCB). Figure 4c shows a photograph of a stretchable neural electrode array with a 50‐µm‐thick PDMS substrate fitting on the visual cortical surface of the left hemisphere of a rat, which indicating a good conformal contact between the electrode array and the curved surface of the rat's brain. The stretchable devices with a 50‐µm‐thick and a 467‐µm‐thick PDMS substrates are selected to compare in ECoG recording. The experiment results are shown in Figure 4d,e, respectively. In order to evaluate the fidelity of ECoG signals obtained by 50‐µm‐thick and 467‐µm‐thick stretchable neural electrode array, the rms amplitude and correlation coefficients of ECoG signals are calculated by Equations S4 and S5 (Supporting Information), which are shown in Tables S1–S4 (Supporting Information). Although the ECoG rms amplitudes of 467‐µm‐thick PDMS neural electrodes are larger than that of screw electrodes, the correlation coefficients of them are smaller than 0.25. The overlarge rms amplitudes and small correlation coefficients demonstrate the low fidelity of ECoG signals. The phenomenon can be interpreted as that obvious environmental noises mixed in the ECoG signals are detected due to the nonconformal contact in neural interface since 467 µm is larger than the calculated critical thickness of 135.5 µm.

On the other hand, the stretchable device with a 50‐µm‐thick substrate performs much better. Not only the ECoG rms amplitudes of the 50‐µm‐thick PDMS neural electrode array are larger than those of the screw electrodes, but also the correlation coefficients of them are larger than 0.78 (shown in Tables S3 and S4 in the Supporting Information). The results indicate that nearly all channels of the 50‐µm‐thick PDMS neural electrodes are in good contact with the brain and the stretchable neural electrode array with a proper thickness can record good ECoG signals.

**Figure 5:**
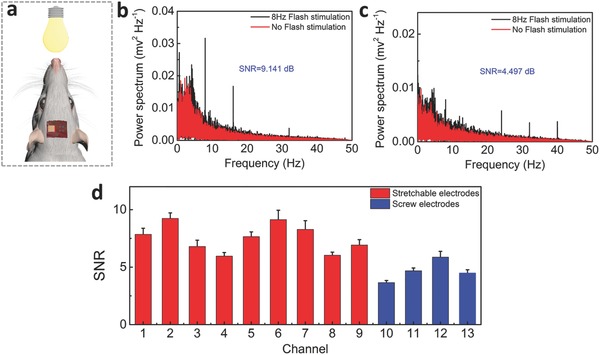
The steady‐state visual evoked potential (SSVEP) of the rat. a) The location of the LED relative to the rat used in detecting SSVEP. b) Power density spectra of the SSVEP recorded over a 3 min time window for stretchable neural electrode array of Ch. 6. c) Power density spectra of the SSVEP recorded by stainless‐steel screw (rigid) electrode of Ch. 13 over the same period. d) Comparisons of the SSVEP SNR in stretchable neural electrode array and the stainless‐steel screw (rigid) electrode at the frequency of 8 Hz.

The steady‐state visual evoked potential (SSVEP) represents a periodic response evoked by a repetitive visual stimulus with a frequency above 4 Hz, which has been widely used in brain–computer interface (BCI) and frequency tagging research.^63^, ^64^ In order to acquire SSVEP, a LED with 3 W in power is fixed between the two eyes of a rat with a distance of 5.6 cm, as shown in **Figure** **5**a. The rat is exposed to flash stimulus with a low square‐wave frequency of 8 Hz. As shown in Figure 5b,c, a clear peak at the expected frequency range can be observed at the power density spectra for both the stretchable and the stainless‐steel screw (rigid) neural electrodes. By quantitatively comparing the performance of different types of electrodes to 8 Hz flash, we find that the SSVEP SNR of the stretchable neural electrode (Ch 6) is higher than that of the stainless‐steel screw neural electrode (Ch 13), implying that the stretchable electrode might exhibit a relatively stronger response to 8 Hz flash. To further check the stability of this observation, we plot the power density spectra of all the channels in Figure S13 of Supporting Information and calculated their corresponding SSVEP SNR (shown in Figure 5d). Consistent with above finding, the results demonstrate that the stretchable electrodes show stronger response to 8 Hz flash than those of stainless‐steel screw electrodes. Therefore, the conclusion can be safely drawed that the stretchable neural electrode array has better SSVEP response.

## 3 Conclusion

In summary, we have demonstrated a thermal release transfer printing method to fabricate stretchable conformal neural electrode array. It is shown that the TRT used in this method can pick up microdevices much easier than a conventional transfer printing stamp due to its large strong to weak dry adhesion strength ratio. A dry adhesive and sacrificial‐layer‐free transfer printing process can be developed for metal/PI based microdevices manufacture. Based on the fracture‐mechanics approach, the peeling velocity dependency and temperature dependency of the energy release rate of this transfer printing method is established by experimental data and theoretical analysis. It is also found that the extent of conformal coverage increases with decreasing elastomeric substrate thickness, which is verified by wrapping experiments on a single cylinder with the PDMS substrates of different thicknesses. The calculated critical thickness for a PDMS substrate with a Young's Modulus of 841 KPa is 135.5 µm for wrapping the brain of rat. In vivo, the surgeries in a rat have demonstrated that the stretchable electrode array with a 50 µm thick substrate maintained perfect contact against the curved surface of the brain in the rat. Further surgeries have indicated that SSEVP responses recorded by the stretchable neural electrode array are more obvious than those detected by stainless‐steel screw electrodes. In the future, thermal release transfer printing method is expected to be useful for the manufacture of other stretchable medical devices including sensor skins, wireless application of bioelectronics, and stretchable health monitoring sensor.

## 4 Experimental Section

*Fabrication of Stretchable Conformal Bioelectronics on Glass*: The TRT (Revalpha, Nitto Denko) was utilized to transfer print microdevices to the ultrathin PDMS (Dow Corning Sylgard 184, fabricate on PTFE culture dish) substrates. The energy release rate was tested by a universal material experiment machine (Shimadzu, Japan).

*Finite Element Analysis*: ABAQUS commercial software was used to study the mechanics properties of the stretchable conformal neural electrode array on the ultrathin elastomeric PDMS substrate. The PDMS substrate was modeled by the hexahedron element (C3D8R), while the PI/Au/PI mesh structure was modeled by the composite shell element (S4R). An ideal elastic–plastic constitutive relation with a Young's modulus of 78 GPa, Poisson's ratio of 0.44, and yield strain of 0.3% describe the mechanical behavior of Au. The Young's modulus and Poisson's ratio are 2.5 GPa and 0.34 for PI.

*ECoG Signals Measurement*: All experiments are approved by the Ethical Committee on Animal Experimentation of the University of Electronic Science and Technology of China (UESTC). Four male Sprague–Dawley rats (body weight around 270 g) were used in the study. All the rats were in narcotism by injecting 1% pentobarbital sodium into cavum abdominis (60 mg kg^−1^). All stereotactic coordinates were relative to bregma with the skull surface flat, according to Paxinos and Watson.^65^ Four small holes were drilled in the skull over primary (secondary) visual cortex (regions potentially involved in SSVEP generation), and drilled vertically to skull surface flat. One small hole was drilled at cerebellum. Stainless‐steel screw electrodes (diameter, 500 µm) were implanted in the drill holes, with the reference position at cerebellum (Cb), which exhibits lower activity compared to other brain sites. The 13‐electrode location is shown in Figure S14 (Supporting Information) and the design of PCB is shown in Figure S15 (Supporting Information). Experiments were performed in a well‐lit and shielded dark room. Before the circular stimulus, the data of a 5 min long control period was recorded for each rat. Next, rats were sequentially exposed to the 8 Hz low frequency stimulus. The voltage of LED is 7 V. ECoG was recorded with a UEA‐FZ amplifier using compatible software developed by our lab (1000 Hz sampling rate), and was filtered using an online bandpass filter between 0.1 and 120 Hz and a 50 Hz notch filter for the line frequency interference. The SSVEP SNR was calculated by Equation S6 of Supporting Information.

## Conflict of Interest

The authors declare no conflict of interest.

## Supporting information

Supplementary

Supplementary
