# Supplementary material for: Thermal Release Transfer Printing for Stretchable Conformal Bioelectronics
Source: Adv Sci (Weinh). 2017 Jul 31;4(11):1700251. doi: 10.1002/advs.201700251 (PMC5700632; doi:10.1002/advs.201700251)
Supplement: Supplementary file 1 — Supplementary [file ADVS-4-na-s001.pdf]

Copyright WILEY-VCH Verlag GmbH & Co. KGaA, 69469 Weinheim, Germany, 2017.

ADVANCED SCIENCE
Open Access

Supporting Information

for *Adv. Sci.,* DOI: 10.1002/advs.201700251

Thermal Release Transfer Printing for Stretchable Conformal Bioelectronics

*Zhuocheng Yan, Taisong Pan, Miaomiao Xue, Changyong Chen, Yan Cui, Guang Yao, Long Huang, Feiyi Liao, Wei Jing, Hulin Zhang, Min Gao, Daqing Guo, Yang Xia, and Yuan Lin**

Copyright WILEY-VCH Verlag GmbH & Co. KGaA, 69469 Weinheim, Germany, 2016.

Supporting Information

Thermal Release Transfer Printing for Stretchable Conformal Bioelectronics

*Zhuocheng Yan, Taisong Pan, Miaomiao Xue, Changyong Chen, Yan Cui, Guang Yao, Long Huang, Feiyi Liao, Wei Jing, Hulin Zhang, Min Gao, Daqing Guo, Yang Xia, Yuan Lin**

The bending stiffness of the thin film

The cross sectional geometry of the thin film on a PDMS backing substrate is illustrated in Figure S7. The PDMS backing substrate has a thickness H and Young's modulus E_{PDMS} = 841 kPa. The distance between the neutral axis and bottom of the thin film is[1]:

$$y0 = \frac{h}{2}\frac{1+\frac{2h_2+h_3}{h}\left(\frac{E_{Au}}{E_{PI}}-1\right)\frac{nb_3h_3}{bh}-\frac{E_{PDMS}H^2}{E_{PI}h^2}}{1+\left(\frac{E_{Au}}{E_{PI}}-1\right)\frac{nb_3h_3}{bh}+\frac{E_{PDMS}H}{E_{PI}h}} \qquad (S1)$$

The bending stiffness of the thin film is

$$EI = E_{PI}bh\left(\frac{1}{3}h^2-hy_0+y_0^2\right)+E_{PDMS}bH\left(\frac{1}{3}H^2+Hy_0+y_0^2\right) + (E_{Au}-E_{PI})nb_3h_3\left[\frac{1}{3}h_3^2+h_3(h_2-y_0)+(h_2-y_0)^2\right] \qquad (S2)$$

For the wrapped state to be energetically favorable,

$$\gamma \geq \gamma_c = \frac{EI}{R^2b} \qquad (S3)$$

where γ is the adhesion energy per unit area. The bottom frame of Figure S7 compares the above relation with a series of experiments. The data, whose error range is ±5%, are consistent with γ~10 mJ m^{-2}, which is comparable to reported values for wet interfaces.

The calculation of RMS amplitude and correlation coefficient

The calculation of RMS amplitude can be developed by

$$x_{rms} = \sqrt{\frac{\sum_{i=1}^{N} x_i^2}{N}} = \sqrt{\frac{x_1^2 + x_2^2 + \cdots + x_N^2}{N}} \tag{S4}$$

Where x_{rms} is RMS amplitude of x sequence, x is a time-related sequence, x_i is the i th element in x sequence, N is sequence length.

The calculation of correlation coefficient can be developed by

$$r = \frac{\sum_{i=1}^{N}(x_i - \bar{x})(y_i - \bar{y})}{\sqrt{\sum_{i=1}^{N}(x_i - \bar{x})^2 \cdot \sum_{i=1}^{N}(y_i - \bar{x})^2}} \tag{S5}$$

Where r is the correlation coefficient of two sequences, x is a time-related sequence, y is another time-related sequence, x_i is the i th element in x sequence, y_i is the i th element in y sequence, N is sequence length, $\bar{x}$ is average value of x sequence, $\bar{y}$ is average value of y sequence.

The calculation of SSVEP SNR

In order to compare the SNRs of the ECoG signals of stretchable neural electrode array and stainless-steel screw electrodes, the SNR of SSVEP signals is defined as

$$SNR = \frac{n \cdot X(f_{stimulus})}{\sum_{f=f_1}^{f_2} X(f)} \tag{S6}$$

Where $X(f)$ is the amplitude spectrum ranged from f_1= 7 Hz to f_2=9 Hz calculated by FFT, $f_{stimulus}$=8 Hz, and n is number of points of $X(f)$ from f_1 to f_2.

Table S1. Comparison of ECoG rms amplitude of the 467-μm-thick PDMS stretchable neural electrode array and the rigid electrodes

Ch	1	2	3	4	5	6	7	8	9	10	11	12	13
RMS	0.2159	0.2188	0.2205	0.2253	0.2293	0.2295	0.227	0.2828	0.2021	0.0764	0.0515	0.067	0.0464

Table S2. Comparison of ECoG correlation coefficient of the 467-μm-thick PDMS stretchable neural electrode array and the rigid electrodes

Ch	1	2	3	4	5	6	7	8	9
10	0.2376	0.2403	0.2286	0.2223	0.2173	0.1391	0.2017	0.1542	0.2401
11	0.1677	0.1707	0.1626	0.1574	0.1526	0.1476	0.1391	0.1017	0.1654
12	0.1435	0.1467	0.1374	0.1337	0.1303	0.1249	0.118	0.0888	0.1457
13	0.0959	0.1004	0.0946	0.0923	0.0899	0.0864	0.0793	0.0594	0.0934

Table S3. Comparison of ECoG rms amplitude of the 50-μm-thick PDMS stretchable neural electrode array and the rigid electrodes

Ch	1	2	3	4	5	6	7	8	9	10	11	12	13
RMS	0.1143	0.1132	0.1016	0.1076	0.1051	0.0941	0.0917	0.0913	0.0943	0.0893	0.0670	0.0896	0.0759

Table S4. Comparison of ECoG correlation coefficient of the 50-μm-thick PDMS stretchable neural electrode array and the rigid electrodes

Ch	1	2	3	4	5	6	7	8	9
10	0.8553	0.8971	0.8703	0.8828	0.9126	0.8843	0.8863	0.9017	0.8761
11	0.7967	0.8144	0.8096	0.8012	0.8098	0.8138	0.798	0.8056	0.7804
12	0.8094	0.859	0.8366	0.8562	0.8947	0.8767	0.8917	0.9072	0.8918
13	0.8194	0.8359	0.8378	0.8341	0.8341	0.866	0.8545	0.8482	0.8342

* The stretchable neural electrode array is with nine channels (Ch 1~9) and the stainless-steel screw electrodes are four channels (Ch 10~13). The maximum and minimum of correlation coefficients are marked in grey.

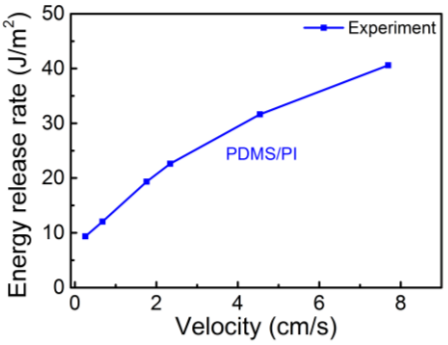

Figure S1. Critical energy release rate versus peel velocity for the PDMS stamp/PI/PMMA/glass substrate.

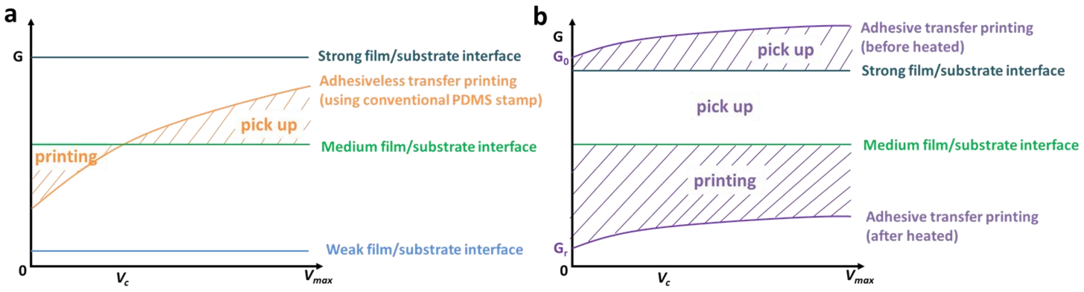

Figure S2. Schematic diagram of critical energy release rates for the film/substrate interface and for the stamp/film interface**.** a) Conventional PDMS transfer printing method. b) Thermal release transfer printing method.

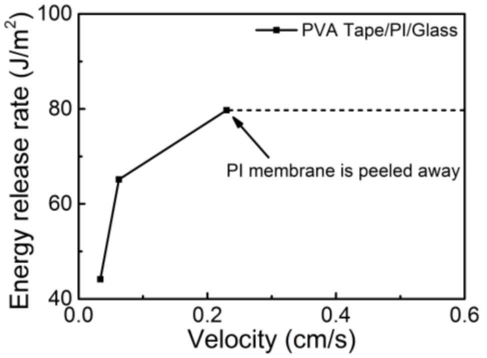

Figure S3. PVA Tape used to test the energy release rate of PI/Glass interface.

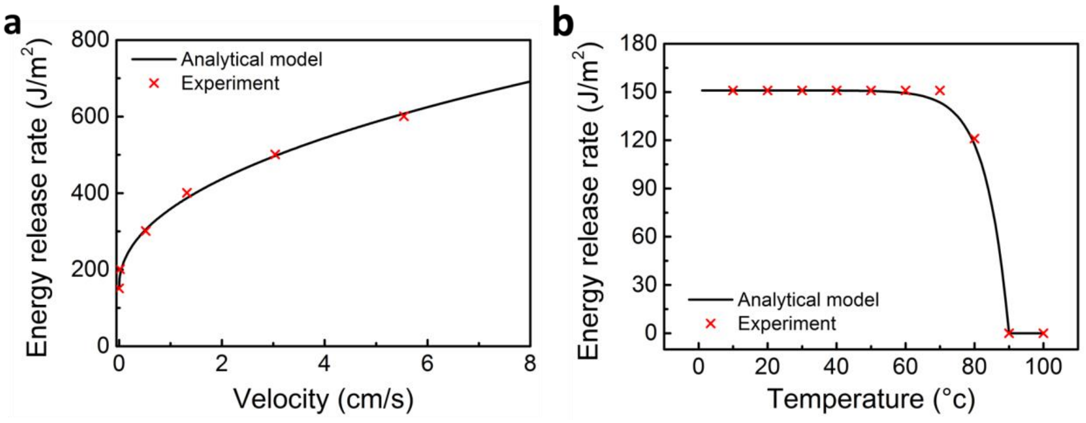

Figure S4. a) Experimental data and theoretical analysis' results of the relationship between the energy release rate and velocity (TRT, PET/PMMA/glass structures). b) Experimental data and theoretical analysis' results of the relationship between the energy release rate and temperature (TRT, PET/PMMA/glass substrate).

The $G_{TRT/PET}$ can also be considered to be determined by two parameters, temperature and peeling velocity. Then, Equation 3 can be further developed as:

$$G_{TRT/PET}(v,T)=\begin{cases}\left(-e^{0.15(T-90)+ln\left(151-\frac{1}{\infty}\right)+151}+151\right)\left(1+(\frac{v}{0.5})^{0.46}\right) & T\leq 90\\ \frac{1}{\infty}\left(1+\left(\frac{v}{0.5}\right)^{0.46}\right) & T>90\end{cases}\qquad (S7)$$

Where the v is current peeling velocity (unit: cm/s), T is current temperature (unit: degree Celsius), e is Euler's constant, $G_{TRT/PET}$ *is* energy release rate of TRT and PET (unit: J/m^2).

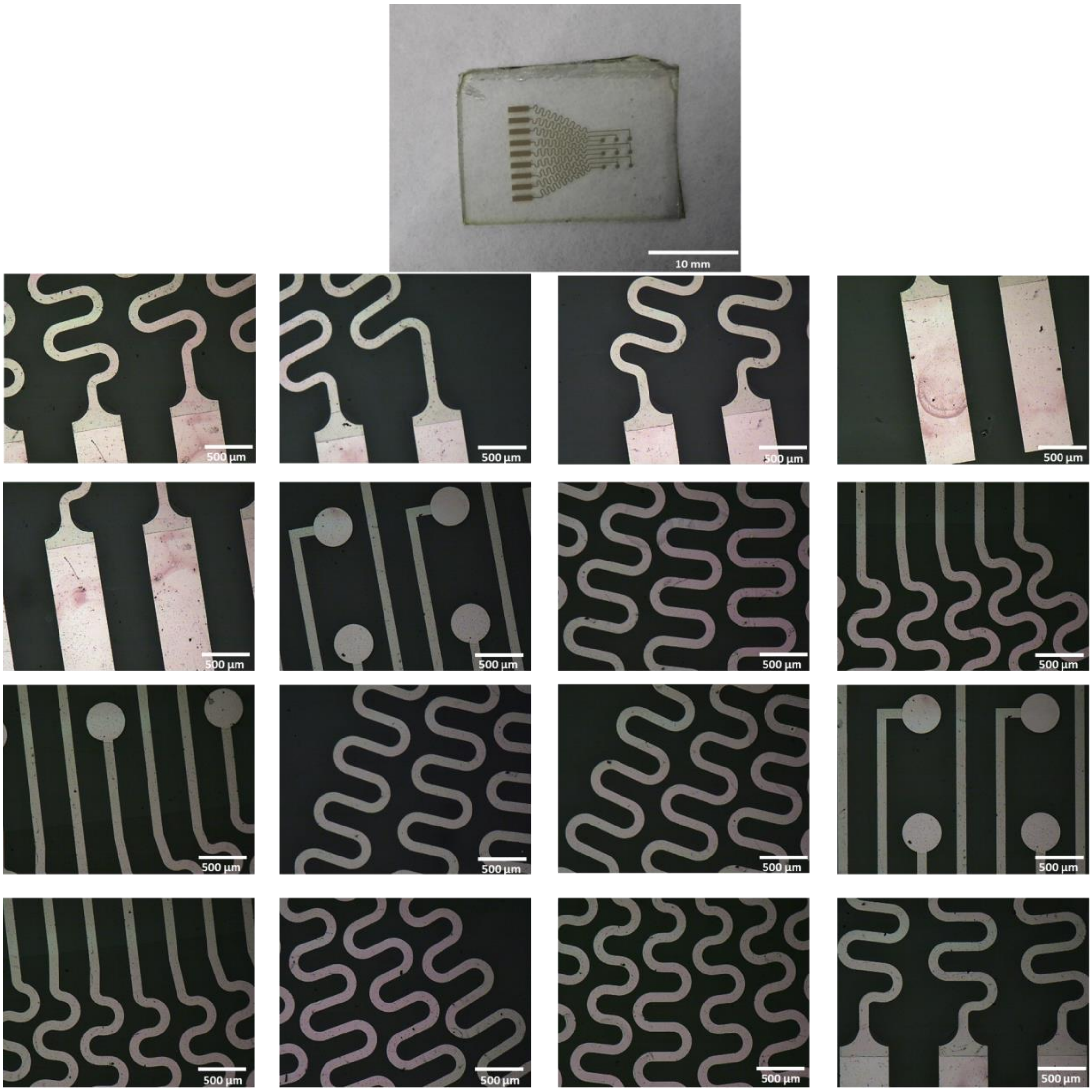

Figure S5. Detailed optical images of the as-fabricated transferable electrode array on a glass substrate (PI/Au/PI/glass).

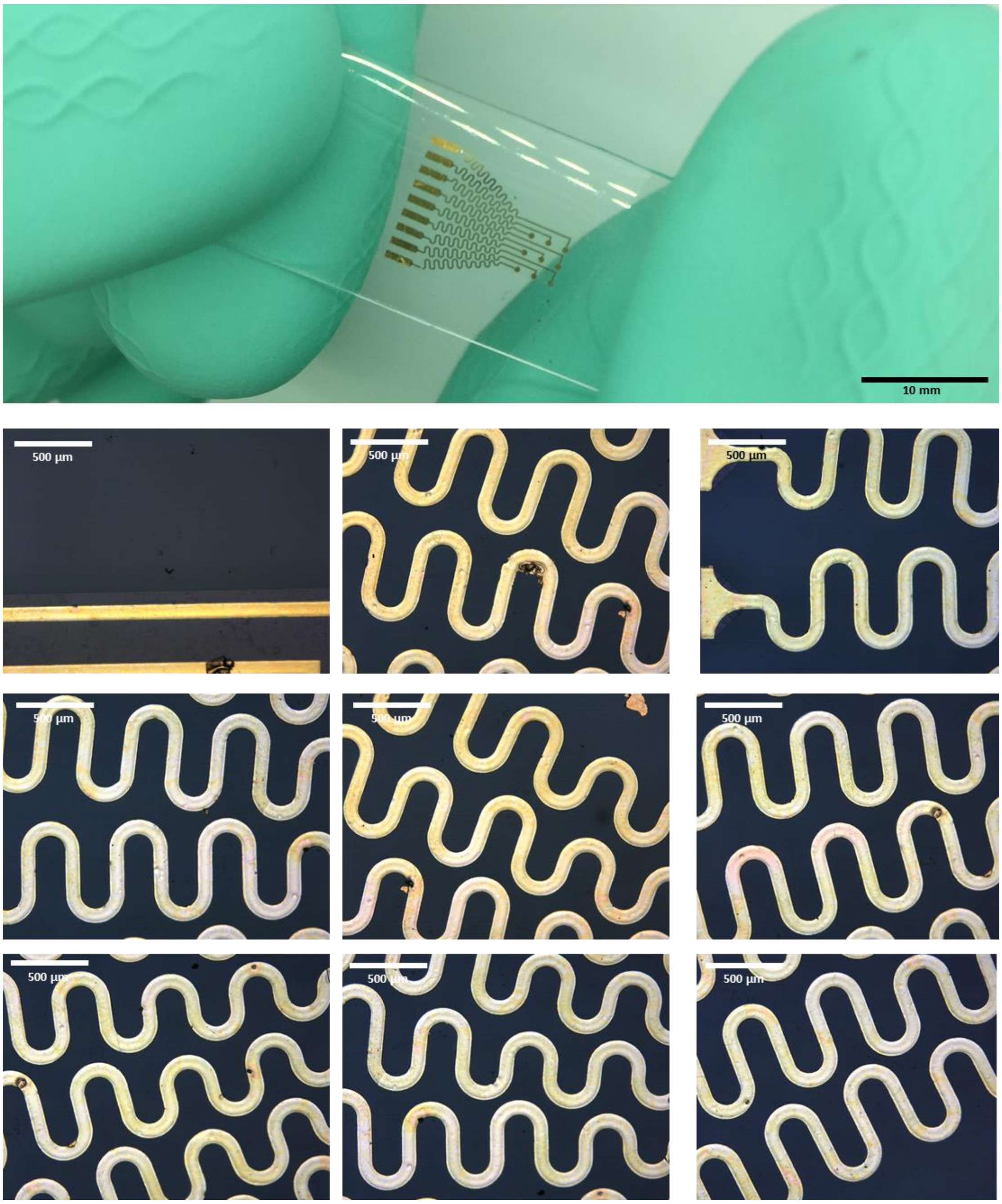

Figure S6 Detailed optical images of the transfer-printed stretchable conformal neural electrode array on a PDMS (50-µm-thick, 841 kPa) substrate.

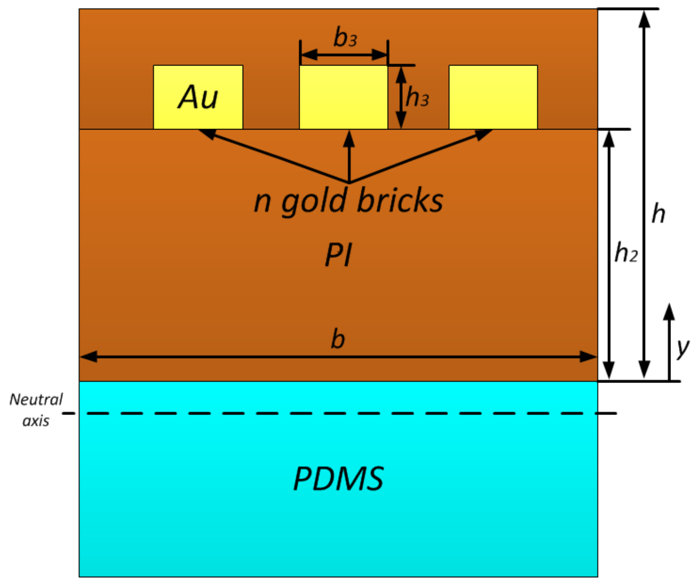

Figure S7. Schematic diagram for analytical model for bending stiffness. Cross section of the stretchable conformal neural electrode array on a PDMS backing substrate, with geometrical parameters illustrated.

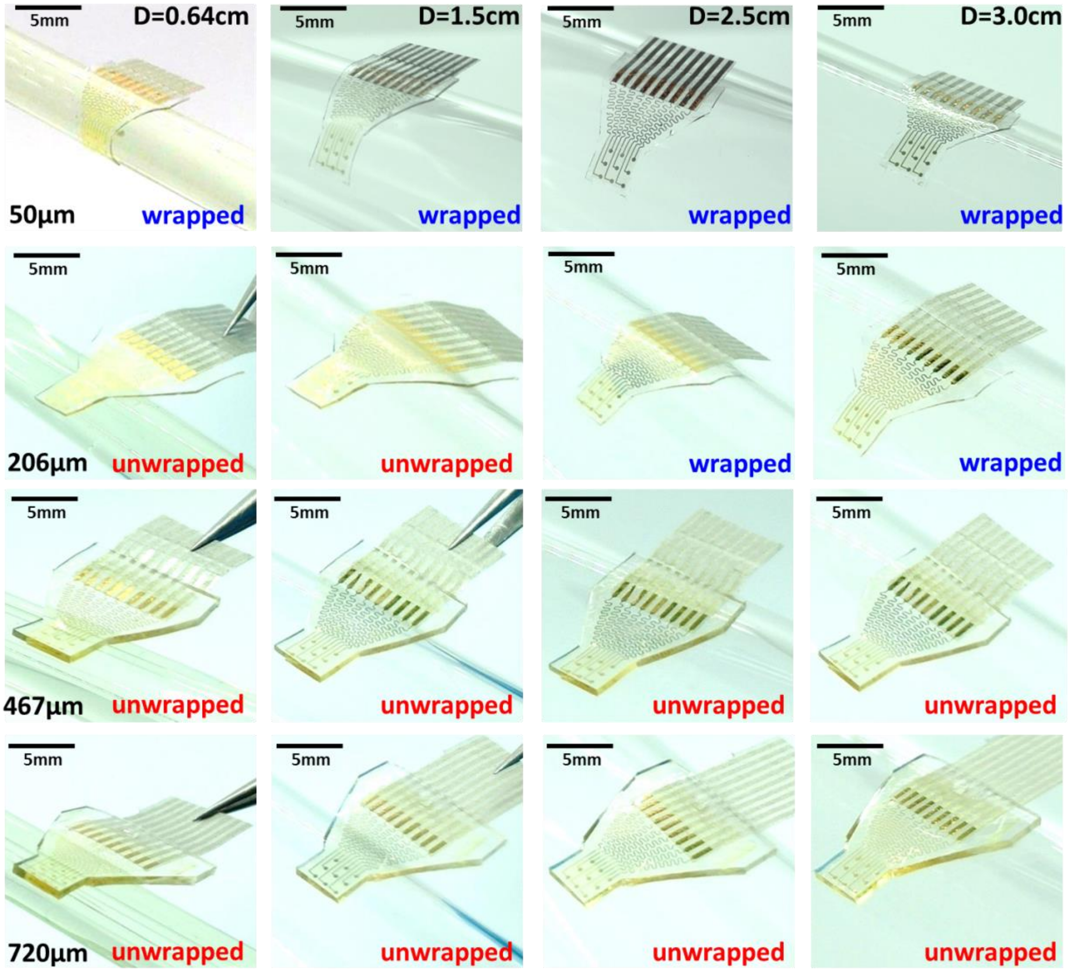

Figure S8. Wrapping experiments on cylinders (whose diameters are 0.64 cm, 1.5 cm, 2.5 cm and 3.0 cm, respectively) using the as-prepared electrode arrays with back elastomeric substrates of different thicknesses (50 μm, 216 μm, 467 μm and 720 μm).

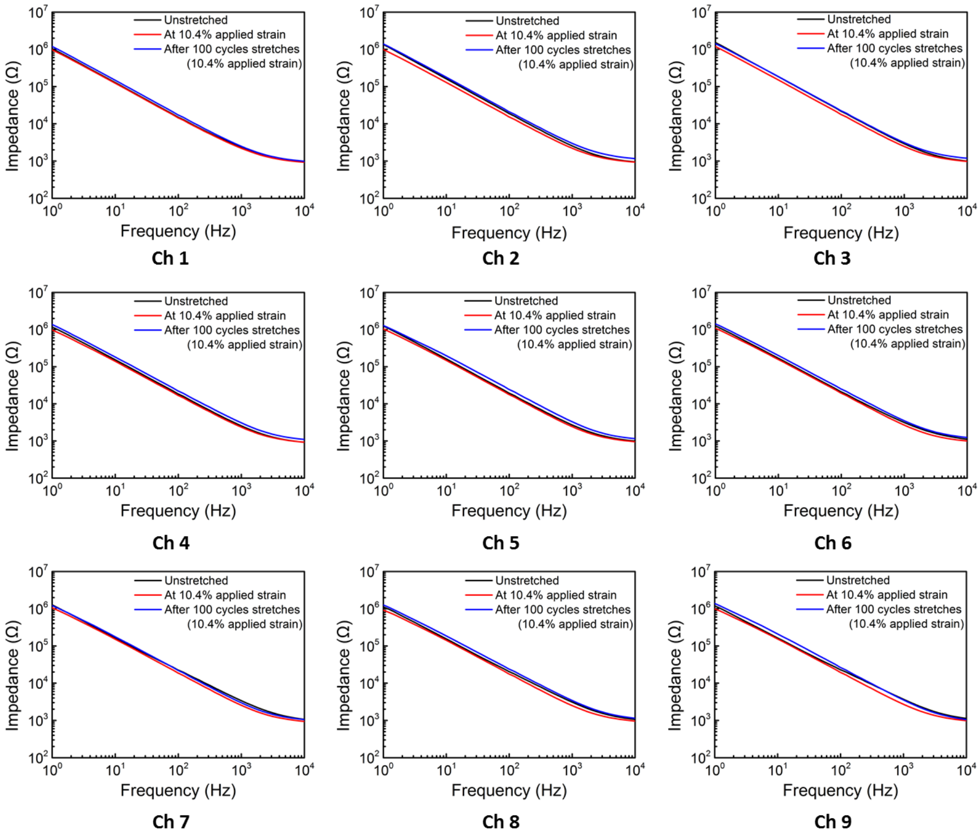

Figure S9. Electrochemical impedance spectra: magnitude measured at nine different recording sites in the stretchable neural electrode array under unstretched, 10.4% applied strain and after 100 cycles of stretches (10.4% applied strain).

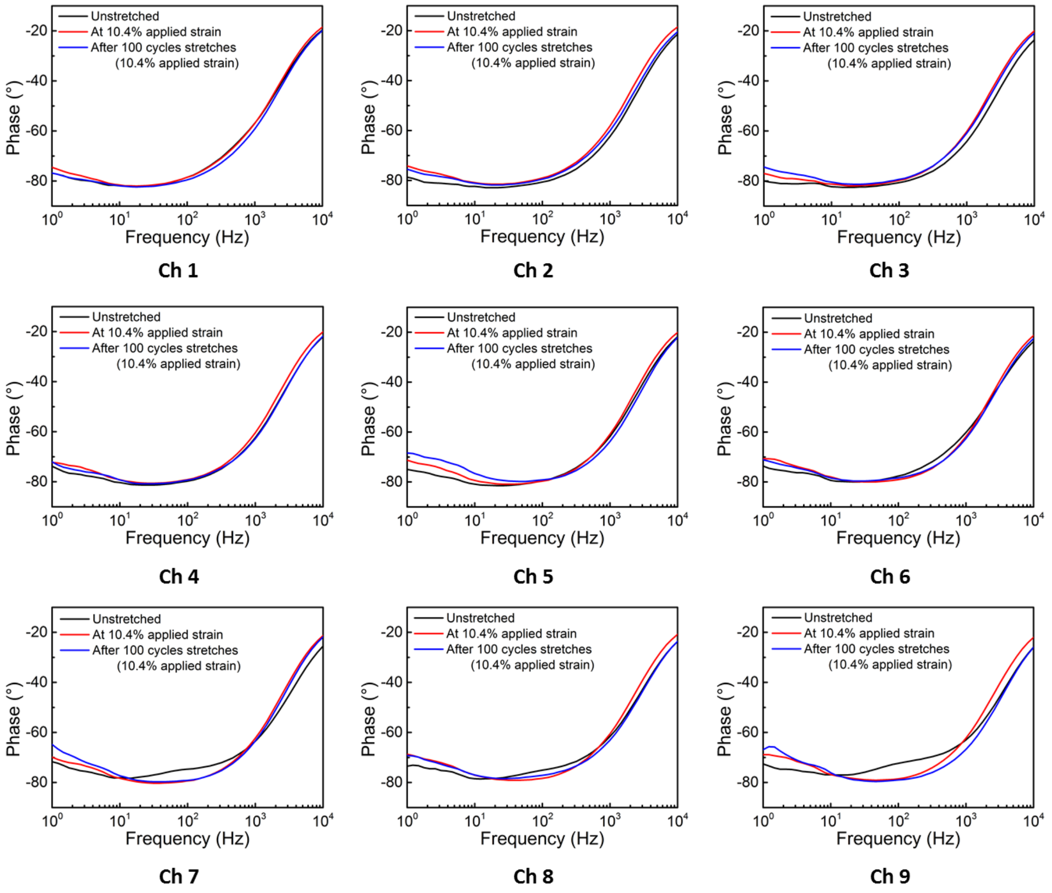

Figure S10. Electrochemical impedance spectra: phase measured at nine different recording sites in the stretchable neural electrode array under unstretched, 10.4% applied strain and after 100 cycles of stretches (10.4% applied strain).

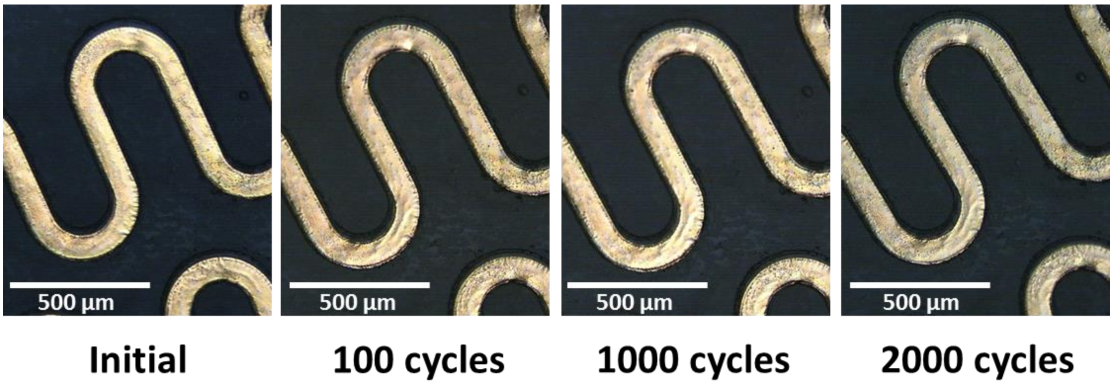

Figure S11. Microscopic images showing cyclic testing results.

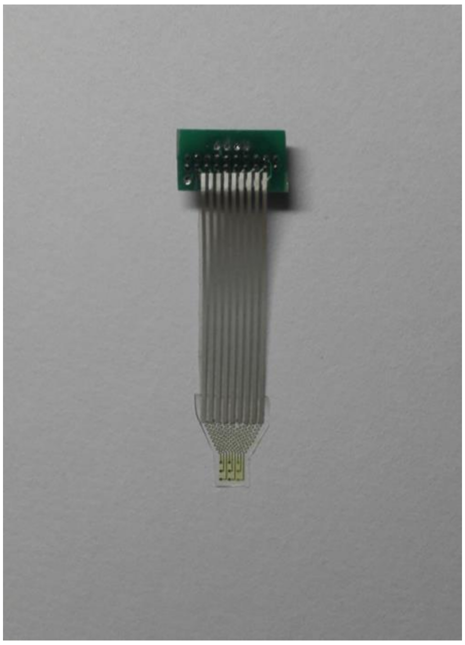

Figure S12. Images of the electrode array after connection of HSC and the printed circuit board.

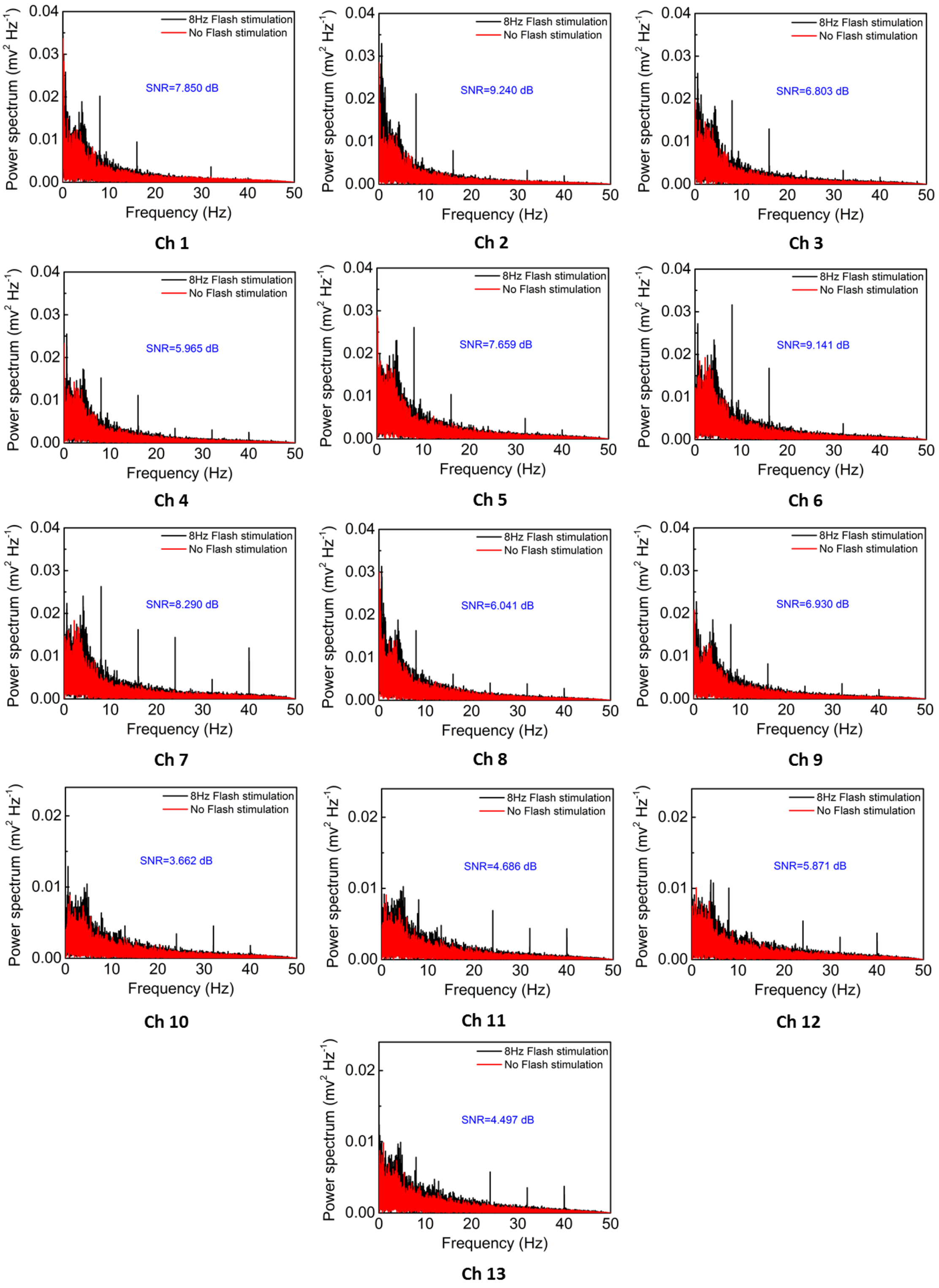

Figure S13. Power density spectra of the SSVEP recorded by 3 min time windows for the 50-um- thick PDMS stretchable neural electrode array (Ch 1~9) and the stainless-steel screw neural electrodes of (Ch 10~13).

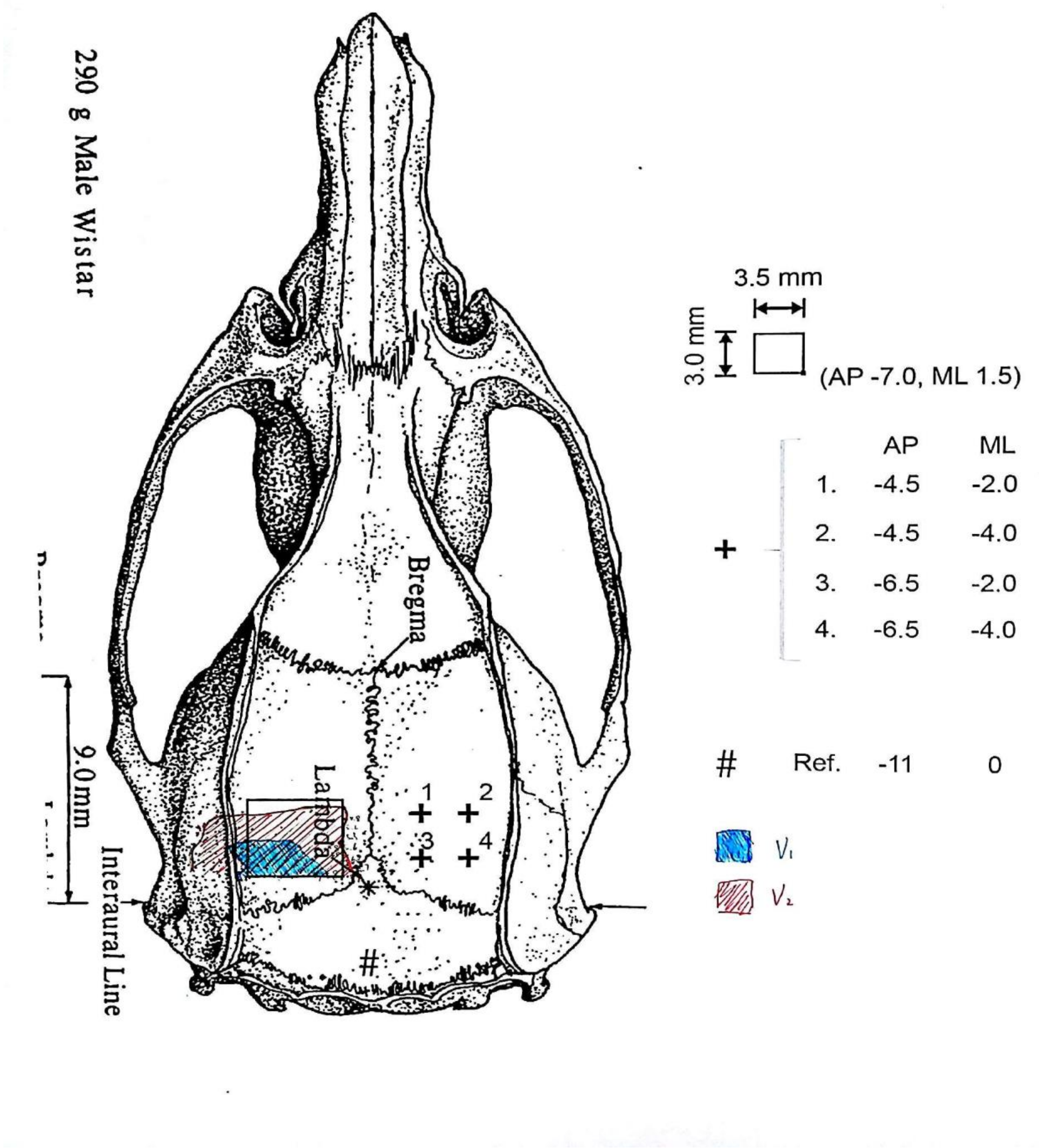

Figure S14. The precise locations of 13-electrode montage[2].

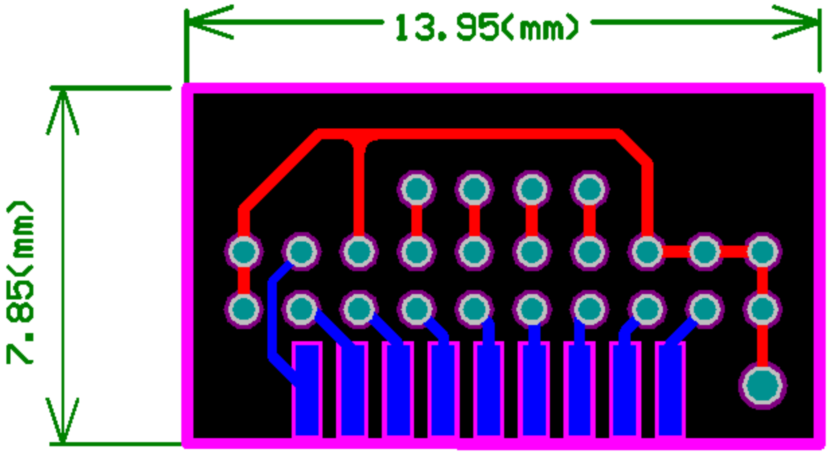

Figure S15. The architecture drawing of the PCB used in the experiments.

References for Supporting Information

[1] D. H. Kim, J. Viventi, J. J. Amsden, J. Xiao, L. Vigeland, Y. S. Kim, J. A. Blanco, B. Panilaitis, E. S. Frechette, D. Contreras, D. L. Kaplan, F. G. Omenetto, Y. Huang, K. C. Hwang, M. R. Zakin, B. Litt, J. A. Rogers, *Nat. Mater.* **2010**, 9, 511.

[2] G. Paxinos, C. Watson, *The rat brain in stereotaxic coordinates*, Boston: Elsevier Academic Press, **2005**
